# Border Patrol Gone Awry: Lung NKT Cell Activation by *Francisella tularensis* Exacerbates Tularemia-Like Disease

**DOI:** 10.1371/journal.ppat.1004975

**Published:** 2015-06-11

**Authors:** Timothy M. Hill, Pavlo Gilchuk, Basak B. Cicek, Maria A. Osina, Kelli L. Boyd, Douglas M. Durrant, Dennis W. Metzger, Kamal M. Khanna, Sebastian Joyce

**Affiliations:** 1 Department of Pathology, Microbiology, and Immunology, Vanderbilt University School of Medicine, Nashville, Tennessee, United States of America; 2 Veterans Administration Tennessee Valley Healthcare System, Nashville, Tennessee, United States of America; 3 Department of Immunology, University of Connecticut Health Center, Farmington, Connecticut, United States of America; 4 Department of Psychology and Human Development, Vanderbilt University, Nashville, Tennessee, United States of America; 5 Center for Immunology and Microbial Disease, Albany Medical College, Albany, New York, United States of America; New Jersey Medical School, UNITED STATES

## Abstract

The respiratory mucosa is a major site for pathogen invasion and, hence, a site requiring constant immune surveillance. The type I, semi-invariant natural killer T (NKT) cells are enriched within the lung vasculature. Despite optimal positioning, the role of NKT cells in respiratory infectious diseases remains poorly understood. Hence, we assessed their function in a murine model of pulmonary tularemia—because tularemia is a sepsis-like proinflammatory disease and NKT cells are known to control the cellular and humoral responses underlying sepsis. Here we show for the first time that respiratory infection with *Francisella tularensis* live vaccine strain resulted in rapid accumulation of NKT cells within the lung interstitium. Activated NKT cells produced interferon-γ and promoted both local and systemic proinflammatory responses. Consistent with these results, NKT cell-deficient mice showed reduced inflammatory cytokine and chemokine response yet they survived the infection better than their wild type counterparts. Strikingly, NKT cell-deficient mice had increased lymphocytic infiltration in the lungs that organized into tertiary lymphoid structures resembling induced bronchus-associated lymphoid tissue (iBALT) at the peak of infection. Thus, NKT cell activation by *F*. *tularensis* infection hampers iBALT formation and promotes a systemic proinflammatory response, which exacerbates severe pulmonary tularemia-like disease in mice.

## Author Summary

NKT cells are innate-like lymphocytes with a demonstrated role in a wide range of diseases. Often cited for their ability to rapidly produce a variety of cytokines upon activation, they have long been appreciated for their ability to “jump-start” the immune system and to shape the quality of both the innate and adaptive response. This understanding of their function has been deduced from *in vitro* experiments or through the *in vivo* administration of highly potent, chemically synthesized lipid ligands, which may not necessarily reflect a physiologically relevant response as observed in a natural infection. Using a mouse model of pulmonary tularemia, we report that intranasal infection with the live vaccine strain of *F*. *tularensis* rapidly activates NKT cells and promotes systemic inflammation, increased tissue damage, and a dysregulated immune response resulting in increased morbidity and mortality in infected mice. Our data highlight the detrimental effects of NKT cell activation and identify a potential new target for therapies against pulmonary tularemia.

## Introduction

The respiratory mucosa is a major site for pathogen entry and hence, requires constant immune surveillance. Like other mucosal surfaces, the lungs are populated by a variety of innate cells and innate-like lymphocytes. One such cell type, the type I, semi-invariant natural killer T (NKT) cells, are enriched within the lung vasculature where they are optimally positioned for early antigen encounter [1]. These pulmonary NKT cells exert diverse functions dependent upon experimental settings [2].

NKT cells express an invariant TCR α-chain (Vα14-Jα18 in mice and Vα24-Jα18 in humans) and one of a restricted set of TCR β-chains and, hence, called semi-invariant. Their name also reflects their hybrid nature, in that they co-express markers of both NK cells and conventional T cells. Their innate-like character is reflected in their ability to rapidly respond to stimulation by producing a wide variety of cytokines [3]. Several subsets of NKT cells have been identified, each of which may have distinct functional consequences in disease conditions where NKT cells are known to play a role [3–7]. NKT cell functions are controlled by microbial or self-glycolipids presented by CD1d molecules or by pro-inflammatory cytokines produced by activated antigen presenting cells (APCs). The quality and magnitude of the NKT cell response is determined by the mode of activation and the chemical nature of the activating lipid(s) [4]. Activated NKT cells can stimulate APCs, natural killer cells, and other leukocytes through the expression of cytokines and costimulatory molecules, thus functioning at the interface between innate and adaptive immunity [4]. Consequently, NKT cells control microbial and tumour immunity as well as autoimmune diseases [8–10].

In the lungs, NKT cells promote inflammation in models of airway hyperreactivity (AHR), acute lung injury (ALI), and chronic obstructive pulmonary disease (COPD) [2]. NKT cells may also contribute to the inflammatory cascade accompanying sepsis, which is often a complication of bacterial infections of the lung [11,12]. In general, pulmonary NKT cells are thought to play a protective role in microbial infections, but in some cases, may also exacerbate disease [2]. However, the mechanisms by which pulmonary NKT cells contribute to disease pathology remain poorly defined. The disparate results encountered in the literature are likely due to the differential function of individual NKT cell subsets, the various means by which NKT cells are activated in different disease settings, and the use of different NKT-deficient mouse models [13,14].

To probe the function of lung NKT cells, we chose a respiratory *Francisella tularensis* infection model as this infection causes lethal pulmonary tularemia. *F*. *tularensis* is a gram-negative facultative intracellular bacterium, which infects multiple cell types including macrophages, dendritic cells, hepatocytes, neutrophils, and epithelial cells [15,16]. The resulting disease targets multiple organs and manifests itself in several forms of differing severity depending on the inciting bacterial strain, dose, and route of infection. Of these, the respiratory route is the most deadly, and the most likely route of infection by weaponized *F*. *tularensis* [17]. After inhalation, patients typically show signs of systemic illness, which may be accompanied by immediate signs of respiratory disease and can result in death in 30–60% of cases if left untreated [18–21]. Although the exact cause of death is unclear, it is likely due to an overwhelming systemic inflammatory response [22]. Mice inoculated intranasally (i.n.) with *F*. *tularensis* fail to mount an effective immune response for the first 48–72h. After this initial immune latency, the response to *F*. *tularensis* is characterized by a robust local and systemic “cytokine storm” reminiscent of sepsis [23,24]. Little is known about the role of NKT cells in pulmonary tularemia—in either humans or mice, due in part to the difficulties in distinguishing them from NK cells, which protect mice from tularemia-like disease-but a beneficial role has been implied [25]. Each of the cell types known to be susceptible to *F*. *tularensis* infection has been shown to activate NKT cells [4]. Therefore, we reasoned that NKT cells might be activated very early after infection and could function in shaping the quality of both the innate and adaptive response.

The results emerging from testing the afore hypothesis revealed that indeed respiratory infection with *F*. *tularensis* activated iNKT cells which produced interferon (IFN)-γ and propagated a sepsis-like proinflammatory response that led to a lethal phenotype in wild type mice. This proinflammatory response was much tempered in CD1d-deficient mice that lacked NKT cells. Strikingly, however, the mutant mice had increased lymphocytic infiltration in the lungs that organized into structures resembling induced bronchus-associated lymphoid tissue (iBALT) at the peak of infection. Hence, NKT cell activation by *F tularensis* infection hampers iBALT formation, which in conjunction with an NKT cell-dependent proinflammatory response, exacerbates severe pulmonary tularemia-like disease in mice.

## Results

### NKT deficient CD1d^-/-^ mice are less susceptible to i.n. LVS infection

IFN-γ is critically required for murine resistance to primary *F*. *tularensis* LVS infection. The early production of IFN-γ after pulmonary LVS infection is primarily attributed to NK cells and double negative T cells [26]. NKT cells are also a source of early IFN-γ and hence, we reasoned that they might contribute to resistance to i.n. LVS infection. We therefore investigated whether NKT cell deficiency would alter disease outcome. Studies of immune function in infectious disease rely on one of two methods—genetic deletion or *in vivo* depletion—by which to assess their contribution to resistance or pathology. Because no unique NKT cell-specific marker has been identified, the available Ab-mediated depletion methods deplete both NK and NKT cells as these two cell types have significant overlap in surface marker expression, making interpretation difficult. Mice made genetically deficient in NKT cells are therefore a better experimental model and were generated previously either by deletion of one of the TCR α-chain gene segments (Jα18^-/-^) or by mutation of the restriction element required for thymic NKT cell selection and antigen presentation in peripheral tissues (CD1d^-/-^) [27–30].

To determine how NKT cell deficiency affects the outcome of pulmonary *F*. *tularensis* infection, we first determined the LD_50_ to i.n. inoculation of the live vaccine strain (LVS) in C57BL/6 (B6) mice using an established method [31,32]. During our preliminary experiments, we found that infected mice lost up to 30% of their initial weight beginning d4 p.i.. Although weight loss was observed at all doses tested (500, 2,000, 8,000 and 30,000 cfu), the degree of disease severity was dependent on the initial inoculum dose. It was only at a dose of 8,000 CFU where we consistently observed moribund mice. At lower doses the majority of mice recovered quickly with few outward signs of disease beyond slightly ruffled fur, which was not consistent between animals. Even at the higher dose, those WT mice that did not succumb by day 12 quickly began to regain weight and appeared otherwise healthy (S1 Fig). Other clinical manifestations included ruffled fur, hunched posture, labored breathing, and reduced mobility. Because we found that weight loss alone was not always an accurate predictor of disease severity, a clinical score based on physical appearance was also included in our endpoint criteria (see Materials and Methods). The resulting LD_50_ for i.n. LVS infection in B6 mice was ~6,000–8,000 cfu (S1 Fig), which was consistent with that previously published by others [33–35].

**Fig 1:**
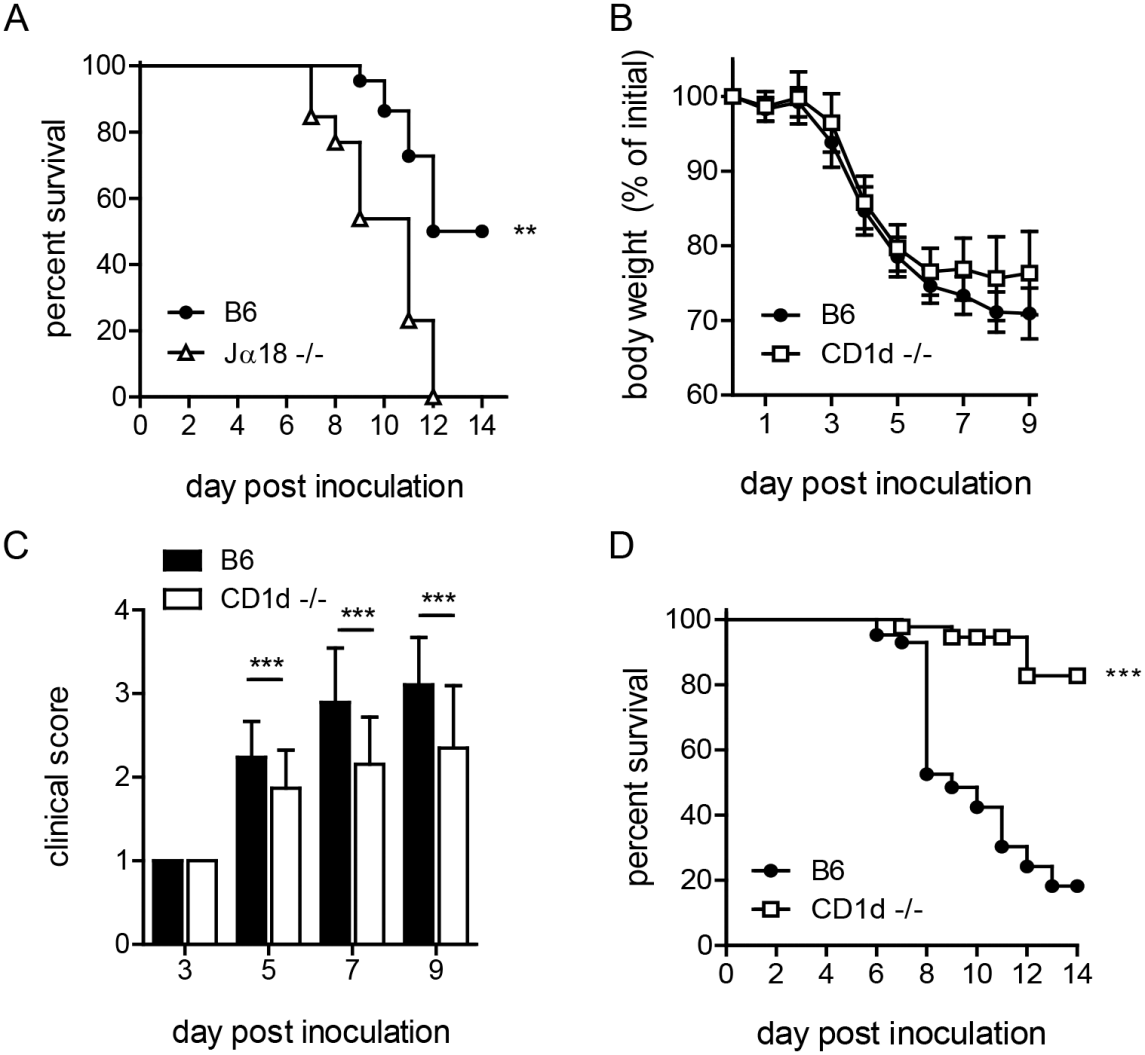
CD1d^-/-^ mice are less susceptible to i.n. LVS infection. **(A)** Survival of B6 or Jα18^-/-^ mice after i.n. LVS infection. Survival of Jα18^-/-^ and WT mice after intranasal LVS infection (8000 cfu). Curves were compared using Log-rank (Mantel-Cox) Test. Data are from one of three similar experiments with at least 10 mice/group. **(B)** B6 or CD1d^-/-^ mice were infected i.n. with LVS and monitored daily for weight loss. The mean (±SD) weight of 50 animals/group in several independent experiments following intranasal infection (Accumulation of five independent experiments with 10 mice/group in each experiment) is presented. **(C)** Clinical score at various time points p.i. as determined in Materials and Methods. Data are cumulative from four similar experiments (mean+SD) with 5–10 mice/group/time point. Data were analyzed as indicated in Materials and Methods. **(D)** Survival of B6 or CD1d^-/-^ mice after i.n. LVS infection. Cumulative survival curves from 4 experiments with 40 mice/group. Curves were compared using Log-rank (Mantel-Cox) Test. **p<0.01, ***p<0.001.

To examine the contribution of NKT cells in resistance to primary pneumonic tularemia, we first tested Jα18^-/-^ mice. Hence, B6 and Jα18^-/-^ mice were inoculated i.n. with ~8,000 cfu LVS and monitored for weight loss and signs of morbidity as described in Materials and Methods. When compared to B6, Jα18^-/-^ mice showed a significant increase in susceptibility to i.n. LVS infection (Fig 1A), initially suggesting a protective role for iNKT cells. Although Jα18^-/-^ mice have historically been used as a model for type I NKT cell deficiency, a recent study demonstrated that they also have a profound defect in conventional αβ T cells, with the loss of an estimated 60% of total TCRα repertoire diversity [36]. This report suggests that the increased susceptibility observed in these mice might be due to this more global T cell deficiency rather than the loss of iNKT cells, since conventional T cells were previously shown to mediate protective immunity to LVS [26,34].

We therefore ascertained the outcome of pulmonary LVS infection in CD1d^-/-^ mice, which have a normal complement of αβ T cells but lack NKT cells. By d4 post-inoculation (p.i.), both B6 and CD1d^-/-^ mice began to show signs of disease, including weight loss and ruffled fur. By d5-6, B6 mice continued to lose more weight and showed more severe outward signs of disease as indicated by clinical score (Fig 1B and 1C). In striking contrast, by d7, nearly all NKT deficient CD1d^-/-^ mice began to recover and a significantly lower percentage of CD1d^-/-^ mice succumbed to i.n. LVS infection (Fig 1D). By d14, fewer than 50% B6 and almost all CD1d^-/-^ mice regained weight and showed no outward signs of disease, surviving the infection (Fig 1D). These data suggest that NKT cells serve a deleterious role in pneumonic tularemia. We therefore reasoned that increased iNKT cell number might further exacerbate disease. Thus, Vα14^tg^ mice, which have increased numbers of iNKT cells [37], were infected intranasally and found to have increased susceptibility as predicted (S2 Fig). Taken together, these data strongly support a detrimental function of NKT cells in pneumonic tularemia and show that CD1d^-/-^ mice are better model than Jα18^-/-^ mice to study the role of NKT cells in disease. Hence, CD1d^-/-^ mice were used for all subsequent experiments.

### Intranasal LVS infection recruits NKT cells to the lung interstitium

**Fig 2:**
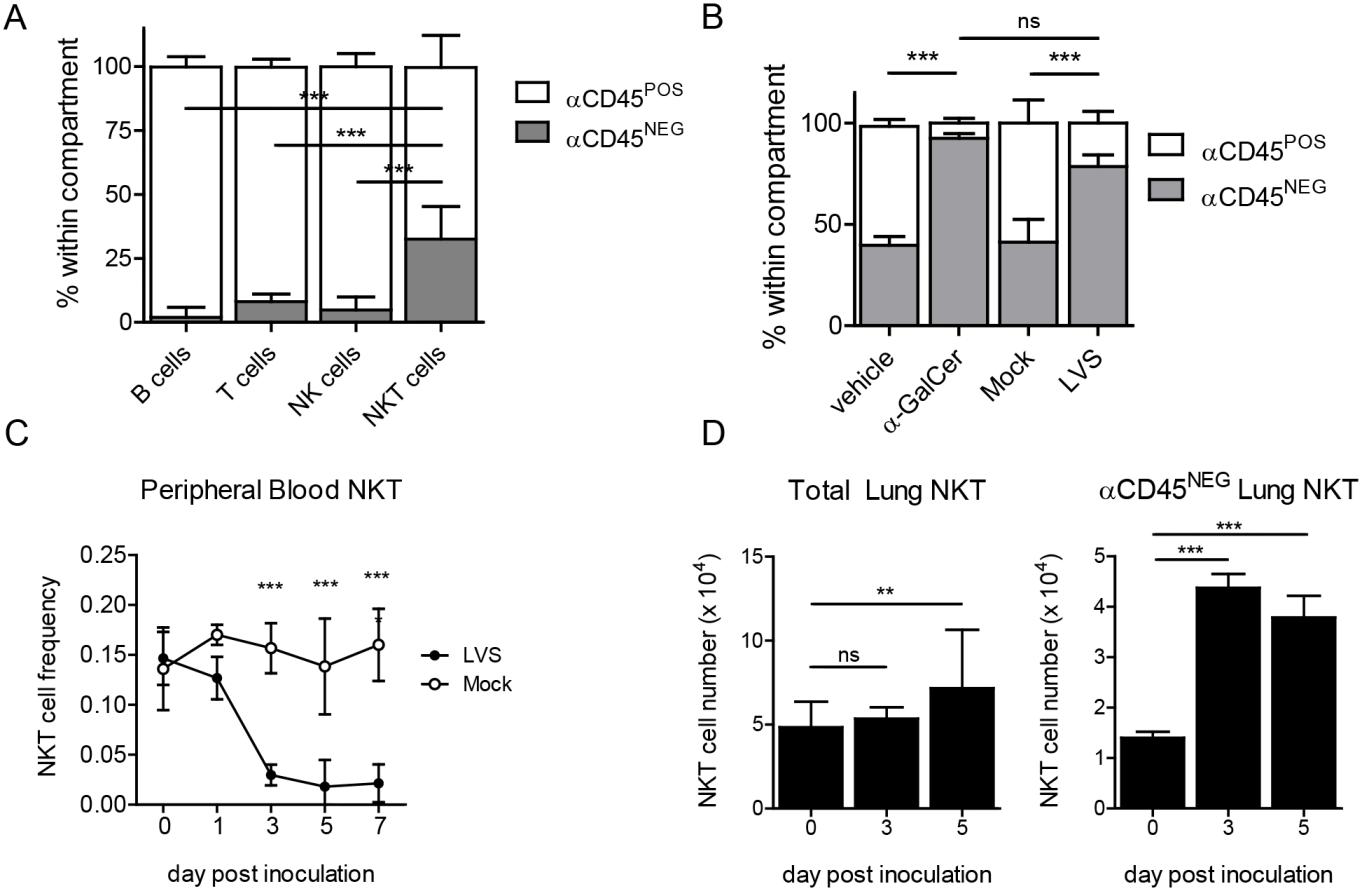
NKT cells are recruited to the lung interstitium in response to LVS infection. **(A)** Intravascular αCD45 Ab staining to determine relative distribution of the indicated lymphocytes in naïve lungs. Data combined from four similar experiments. Bars are mean+SD (*n* = 13 mice). Interstitial NKT cell frequency was compared to all other lymphocyte populations as described in Materials and Methods. **(B)** Frequency of lung NKT cells among B220^-^ cells after i.n. αGC treatment or LVS inoculation. Mean+SD from one of two similar experiments (*n* = 3–4 mice/group). Frequency of interstitial NKT cells was compared by one-way ANOVA with Tukey’s posttest. **(C)** Frequency of NKT cells in peripheral blood of LVS-infected or mock-infected mice. Mean±SD from one of two similar experiments (*n* = 3–4 mice/group). Comparison was made by two-way ANOVA with Bonferroni posttest. **(D)** NKT cell number in total lung or lung interstitium after i.n. LVS infection. Mean+SD combined from two similar experiments (*n* = 7 mice/ time point). Day 3 and day 5 post inoculation were compared to naïve mice as described in Materials and Methods. *p<0.05, **p<0.01, ***p<0.001.

NKT cells are overrepresented among T cells in the healthy lung compared to their frequency in other organs. These tissue-resident NKT cells do not recirculate but rapidly extravasate into interstitial spaces upon recognition of lipid antigens such as the potent CD1d-restricted agonist α-galactosylceramide (αGC) [1,38]. Whether NKT cells behave similarly in response to bacterial infection, where lipid ligands are presumably of lower affinity [4], is unknown. To visualize the location of NKT cells in naïve lungs, we injected B6 mice with anti-CD45 antibody (αCD45 Ab) i.v., allowed it to circulate through blood for several minutes, and tracked the anatomic localization of lung mononuclear cells by flow cytometry after labeling lung leukocytes with Abs against lineage-specific markers. This quick procedure allows circulating αCD45 Ab to label all intravascular cells (αCD45^POS^), while cells that are in the tissue interstitial space would be αCD45^NEG^. We confirmed that the largest percentage of lymphocytes (T, B, and NK cells) was in the αCD45^POS^ intravascular population as reported previously [39]. Interestingly, we found that a proportionately higher frequency of pulmonary NKT cells were present in the interstitium (αCD45^NEG^) when compared to other lymphocytes (Fig 2A and S3 Fig). Such localization suggests that pulmonary NKT cells occupy a frontline niche that allows them to rapidly sense and respond to inhaled antigens.

To test the hypothesis that pulmonary NKT cells rapidly engage in host defense at frontline niches, we inoculated B6 mice i.n. with ~LD_50_ of LVS. On d3 p.i., we injected αCD45 Ab and, after several minutes, lungs were analyzed by flow cytometry for NKT cell localization. We found a significant increase in the frequency of αCD45^NEG^ interstitial NKT cells that was equivalent to the frequency observed after i.n. αGC treatment (Fig 2B and S3 Fig). Furthermore, beginning d3 after i.n. LVS inoculation, we found a significant decrease in the frequency of NKT cells in the blood when compared to uninfected controls (Fig 2C and S3 Fig). This reduction in circulating NKT cells was coincident with their increased numbers in the lungs (Fig 2C and 2D), more specifically and significantly in the lung interstitium (αCD45^NEG^ lung NKT). Thus, i.n. LVS infection rapidly recruits NKT cells from the vasculature into the infected lung interstitium. This finding is consistent with a previous report showing NKT cell extravasation into the lung interstitium after respiratory αGC administration [1].

### A putative LVS-derived antigen activates lung-resident NKT cells through the T cell receptor

**Fig 3:**
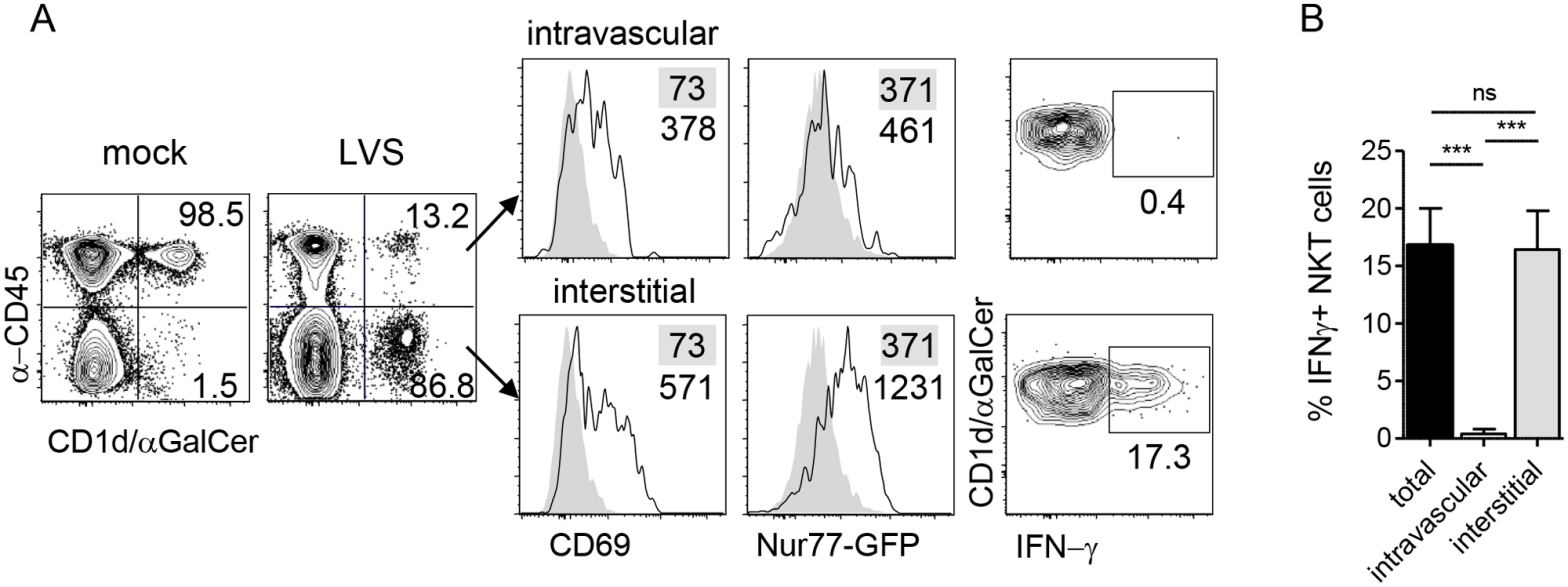
NKT cell activation by LVS is TCR-dependent. **(A)** Representative plots of NKT cell localization, activation, and IFN-γ production after i.n. LVS infection of Nur77^gfp^ mice. Numbers in contour plots are % NKT cells. Numbers in histograms are median fluorescence of infected mice (open histograms) and mock-infected controls (shaded histograms). Data are representative of three similar experiments (*n* = 10). **(B)** Percent IFN-γ+ NKT cells in each compartment after LVS infection. Bars are mean+SD. Data are representative from one of three similar experiments (*n* = 3 mice). Comparison was made by one-way ANOVA with Tukey’s posttest. ***p<0.001.

NKT cells can be activated by recognition of CD1d-bound microbial lipids or self-lipids presented by activated APCs, or by cytokines which can activate these cells independent of TCR stimulation [4]. The mechanism by which NKT cells are activated can influence their function. To determine whether the accumulating NKT cells were activated by LVS infection, we monitored expression of the activation marker CD69 which reports on NKT activation in general, and we employed Nur77^gfp^ reporter mice, which increase GFP expression only upon NKT cell activation via CD1d-restricted microbial glycolipids but not self-lipids [40]. On d3 post i.n. LVS inoculation, we found that both interstitial and intravascular NKT cells increased CD69 expression (Fig 3A). Strikingly, however, only the interstitial population showed increased GFP fluorescence (Fig 3A). This result indicated that local LVS antigen presentation occurred within the interstitium to activate LVS-recruited NKT cells *in situ*.

To determine the consequence of interstitial NKT cell activation, intracellular cytokine staining was conducted directly *ex vivo* without any restimulation. We found that the GFP^hi^ interstitial NKT cells but not GFP^lo^ intravascular cells produced IFN-γ (Fig 3A and 3B), demonstrating that this cytokine production was a result of LVS infection *in vivo*. Taken together, these data suggest that upon i.n. *F*. *tularensis* LVS infection, NKT cells migrate from the periphery and accumulate within the infected lung where they are activated through their TCR to produce IFN-γ.

### Modestly lower bacterial burden correlates with better clinical score in CD1d^-/-^ mice upon pulmonary LVS infection

**Fig 4:**
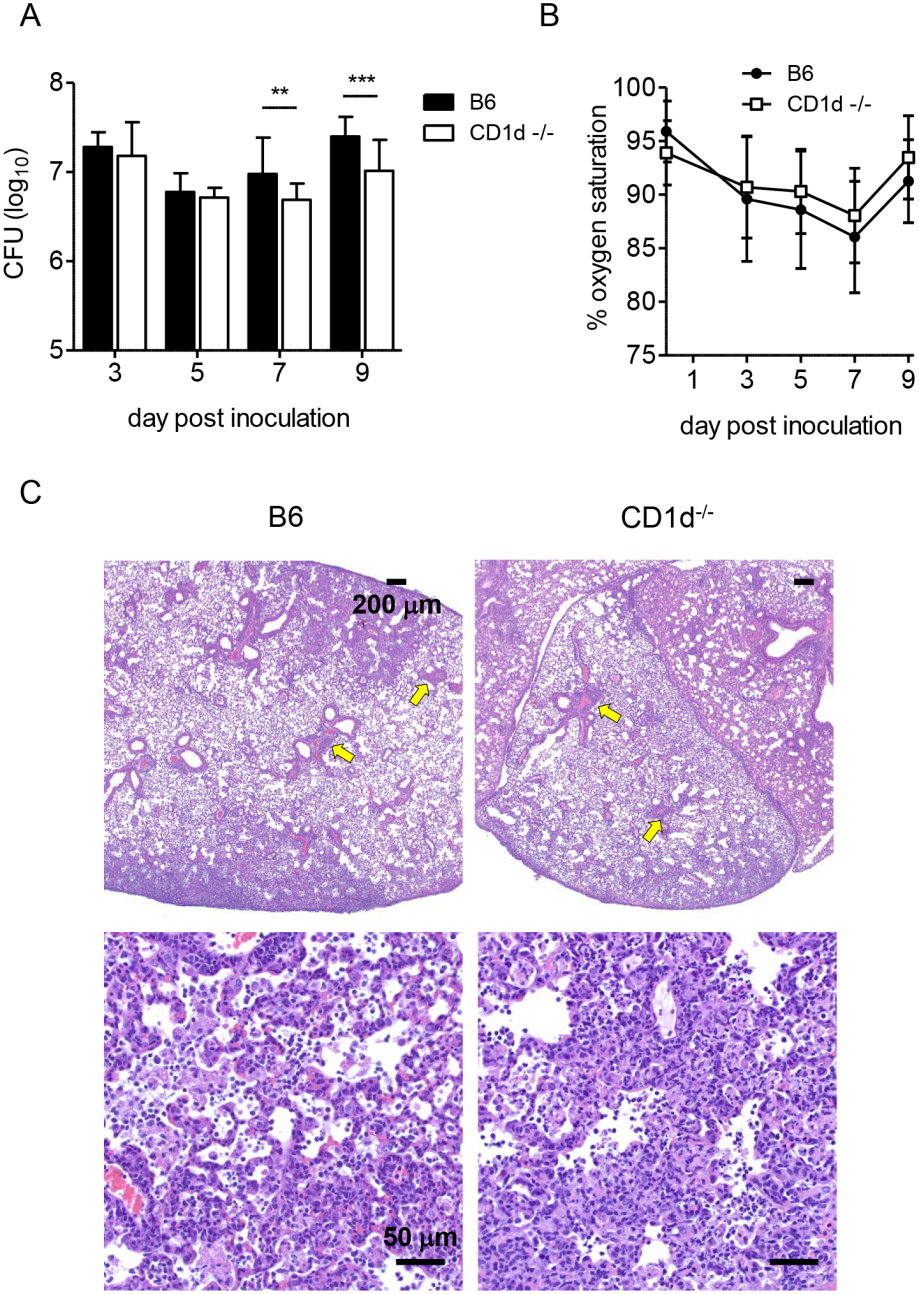
CD1d^-/-^ mice have modestly reduced lung LVS burden but no differences in lung pathology. **(A)** Lung burden was determined at various times p.i. as described in Materials and Methods. Data are combined from at least 3 separate experiments. Bars are mean+SD of 10–15 mice/group/time point. **(B)** Oxygen saturation was measured as described in Materials and Methods. Data are cumulative of 3 separate experiments with 5–10 mice/group (mean±SD). **(C)** H&E stained formalin fixed paraffin embedded (FFPE) lung sections d9 p.i. 40X (top) or 400X (bottom) magnification with scale bars as indicated. Data are representative of three mice per group. Arrows indicate aggregations of lymphocytes. Data were analyzed as indicated in Materials and Methods. **p<0.01, ***p<0.001.

Two factors can affect overall fitness after infection: pathogen burden and the level of organ pathology. To determine whether the decreased susceptibility of CD1d^-/-^ mice to LVS was due to differences in bacterial burden, we measured LVS burden in lung homogenates at various time points post i.n. LVS inoculation. Lung burden was similar in both groups, with a modest but statistically significant difference (two-fold) seen only at d7 and d9 in infected CD1d^-/-^ mice (Fig 4A), which was concomitant with the observed differences in clinical score and weight loss (Fig 1B and 1C).

Since the observed differences in illness began to appear at d7 p.i., we focused on this time point for subsequent analyses. Differing degrees of lung pathology can result in different animal fitness despite similar bacterial burden [35,41,42]. To determine whether the difference in morbidity was due to differential lung damage, we measured blood oxygen saturation (SpO_2_), which was suggested as an accurate measure of lung pathology [43]. While both groups of mice showed decreased SpO_2_ levels, there were no significant differences observed between groups (Fig 4B). To more directly assess tissue damage, H&E-stained lungs were evaluated at d7 and d9 p.i. As predicted by pulse oximetry data, the findings in the lungs of B6 and CD1d^-/-^ mice were similar. Lungs from both groups of mice displayed focally extensive interstitial pneumonia with perivascular cuffs of mononuclear cells. Inflammatory infiltrate consisting of macrophages and neutrophils filled the alveoli, which also contained necrotic debris and edema with focal areas of necrosis on the alveolar walls (Fig 4C).

**Fig 5:**
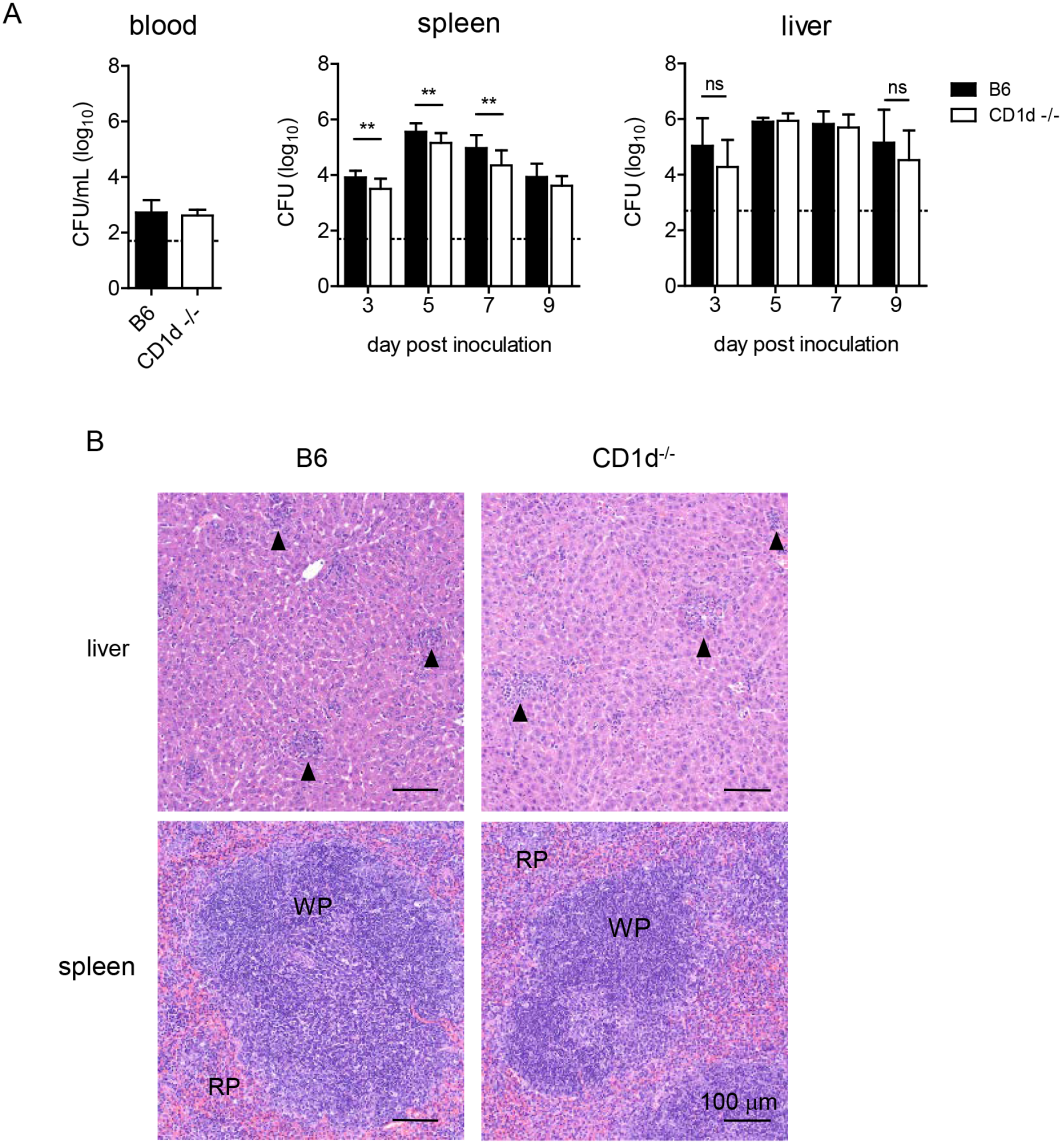
CD1d^-/-^ mice have reduced splenic LVS burden but no difference in the liver. **(A)** Blood, liver, and spleen burden were determined as in Fig 4. Data are combined from 3 separate experiments. Bars are mean+SD of 6–10 mice/group/time point. Data were analyzed as indicated in Materials and Methods. **p<0.01. **(B)** H&E stained FFPE liver (top) and spleen (bottom) sections d9 p.i., 200X magnification. Data are representative of 3 mice/group. Arrows indicate granulomas.

After i.n. infection, *F*. *tularensis* rapidly disseminates to the periphery [44]. The kinetics and extent of dissemination are suggested as determinants of disease severity [45–48]. Hence, we measured burden in blood, liver, and spleen. LVS was only transiently detectable in the blood, where levels peaked at d3 p.i., but there were no differences in bacteremia between groups (Fig 5A). Liver burden was similar in both groups, but CD1d^-/-^ mice had significantly lower splenic burden d3–7 p.i. (Fig 5A). Further analysis showed that only lung burden—but not liver or spleen—correlated with weight loss at d7 p.i. (S4 Fig). Consistent with these findings, histopathological analysis failed to identify any striking differences in either liver or spleen pathology after intranasal inoculation (Fig 5B). The extent of hepatic granuloma formation did not differ between groups (Fig 5B). Contrary to previous reports in BALB/c mice [49,50], splenic architecture was mostly intact with some evidence of apoptosis and extramedullary hematopoiesis that did not seem to differ between groups (Fig 5B).

In summary, the above data indicate that the modest differences in bacterial burden in the lungs and peripheral organs, or another mechanism, but not differential pulmonary, hepatic, or splenic histopathology could explain the milder disease observed in NKT cell deficient mice.

### Increased lymphocyte infiltrates organize into tertiary lymphoid structures in CD1d^-/-^ lungs in response to LVS infection

**Fig 6:**
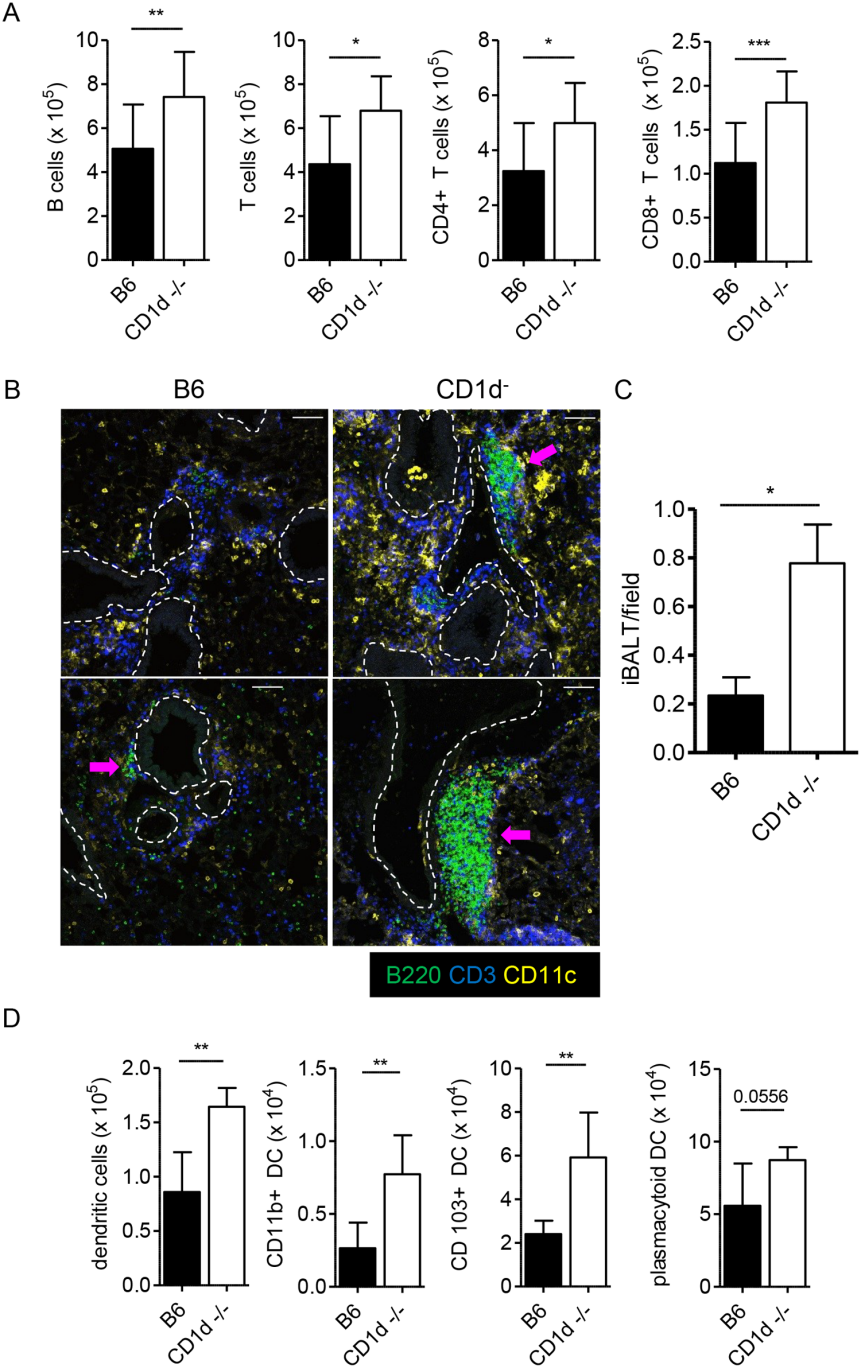
Formation of iBALT in CD1d^-/-^ mice after i.n. LVS infection. **(A)** Groups of B6 or CD1d^-/-^ mice were infected i.n. with LVS. On d7 p.i., lungs were analyzed by flow cytometry for the indicated cell populations. Data are cumulative of 2 experiments with 9–10 mice/group. Bars are mean+SD; compared as indicated in Materials and Methods. **(B)** Representative images from *F*. *tularensis* LVS-infected lungs d7 p.i. Images are individual mice from 2 of 3 similar experiments. 20X magnification; bars are 80 μm. Dashed lines indicate large airways as identified by autofluorescence of epithelial cells. Arrows indicate rudimentary iBALT formation in B6 lung and well-developed iBALT in lungs of CD1d^-/-^mice. **(C)** Enumeration of iBALT from d7 infected lung sections. Data are cumulative from three experiments (*n* = 6 mice/group). Bars are mean+SD. Data were compared using Mann Whitney U test. **(D)** DC subsets in infected lungs were identified as described in Experimental Procedures. Representative results from 1 of 3 similar experiments (*n* = 5 mice/group). Bars are mean+SD; Mann Whitney U test. *p<0.05, **p<0.01, ***p<0.001.

The differences described thus far become most pronounced at d7 p.i, suggesting that the quality of the adaptive response may be the principal underlying cause of the reduced susceptibility observed in CD1d^-/-^ mice. We therefore analyzed lymphocyte numbers in the lungs after LVS infection. Lungs of CD1d^-/-^ mice had consistently higher numbers of both B and T lymphocytes present at d7 p.i. (Fig 6A). To directly visualize the localization of these cells—whether the lymphocytic infiltration was in the pulmonary vasculature or had extravasated into the lung tissue—fixed frozen sections of infected lung were stained and examined by confocal microscopy. Consistent with earlier analysis of H&E stained sections, both groups of mice showed extensive perivascular and peribronchiolar infiltration of leukocytes. Significantly however, in addition to an increased cellularity in CD1d^-/-^ mice, there was a striking difference in the degree of organization of the infiltrating immune cells. We found evidence for the formation of tertiary lymphoid structures, iBALT, within the infected lungs of CD1d^-/-^ mice (Fig 6B). These structures were marked by the formation of B cell follicles, which were surrounded by T cell congregates. In contrast, fewer iBALT structures were formed in B6 mice by d7 p.i. (Fig 6C) and those that developed were small and appeared rudimentary in that T and B cells were scattered or at best loosely packed together (S5 Fig). Such tertiary lymphoid structures were not observed in uninfected CD1d^-/-^ or B6 mouse lungs (S6 Fig) suggesting that iBALTs formed in response to pulmonary LVS infection.

Dendritic cells (DCs) are both necessary and sufficient for the induction of iBALT within the lungs [51]. Consistent with previous reports, concentrations of CD11c^+^ cells were also observed in and around the B and T cell zones of iBALT formed in CD1d^-/-^ lungs (Fig 6B). Further characterization of CD11c^+^ cells within the infected lungs by flow cytometry revealed that all DC subsets identified were increased in the LVS infected lungs of CD1d^-/-^ mice (Fig 6D). Considering that DCs have been implicated as a primary vehicle for dissemination of *F*. *tularensis* from the infected lung [45], this finding may also partially explain the reduced splenic burden in CD1d^-/-^ mice. Hence, iBALT formation in response to LVS infection of the lungs is associated with milder tularemia-like disease in mice and prognosticates recovery.

### Decreased neutrophilia in CD1d^-/-^ mice after LVS infection

**Fig 7:**
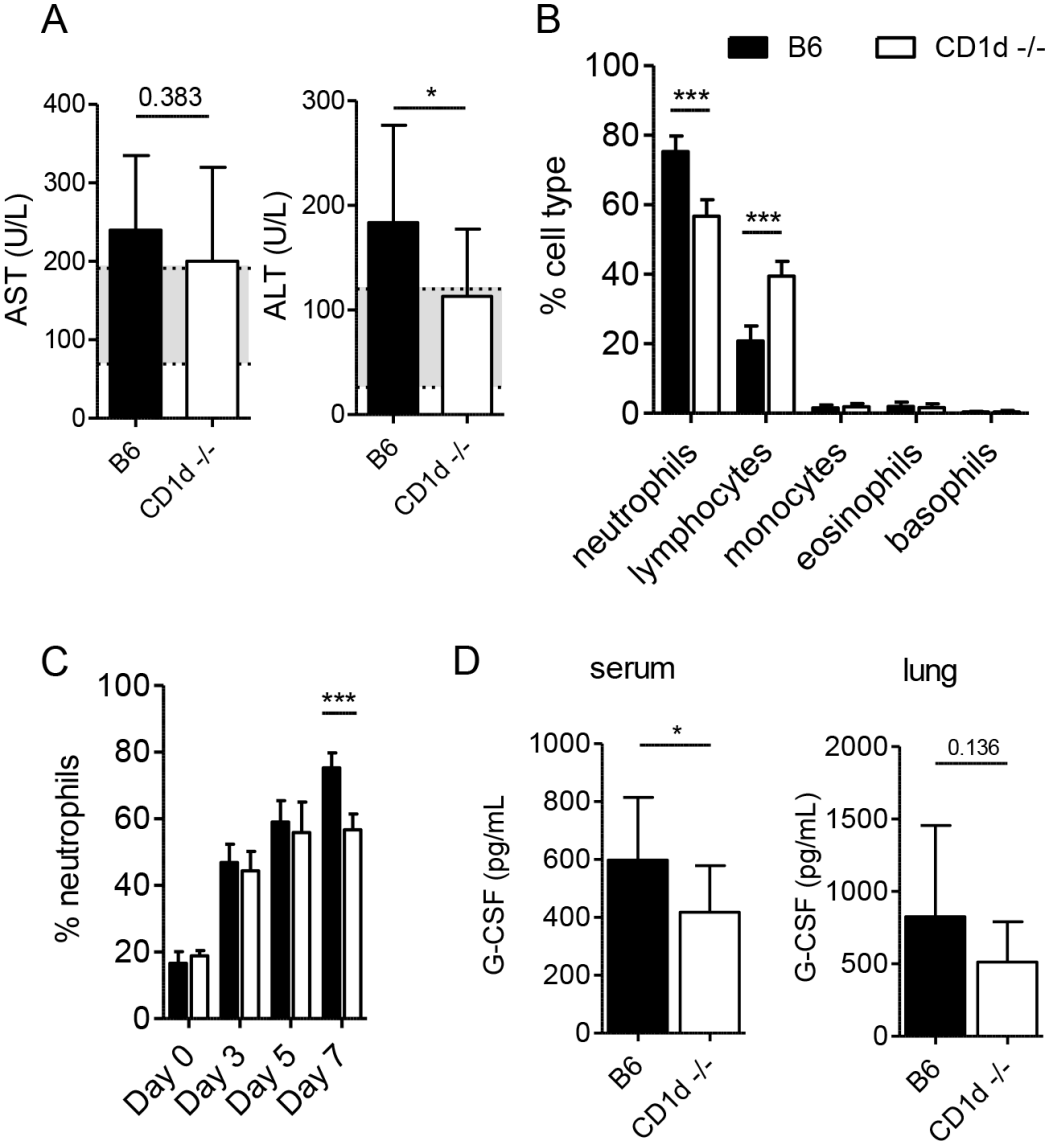
CD1d^-/-^ mice have less severe neutrophilia after intranasal LVS infection. **(A)** AST and ALT levels were measured in serum of infected mice on d7 p.i. Data are combined from 3 experiments (*n* = 10–12). Mean+SD. Shaded regions denote reference range. **(B)** CBC analysis of blood from LVS-infected mice performed on d7. Data are combined from 2 separate experiments (*n* = 15 mice/group). Bars are mean+SD. **(C)** Percent neutrophils in blood at the indicated time points p.i. Data are combined from 2 separate experiments. Bars are mean+SD of 5–15 mice/group/time point. **(D)** On d7 serum and lung G-CSF levels were measured by CBA. Data are combined from 2 experiments (*n* = 15 mice/group). Bars are mean+SD. Comparisons were made as indicated in Materials and Methods. *p<0.05, ***p<0.001.

A previous study showed that increased disease severity in a murine tularemia model was associated with hepatic damage and neutrophilia [52]. NKT cells activated by LVS could cause liver damage through direct lysis of infected hepatocytes [53,54]. They might also promote neutrophilia through production of granulocyte colony-stimulating factor (G-CSF), the major neutrophil survival and proliferation factor [55]. Thus we monitored these two disease parameters in CD1d^-/-^ mice. Although histological analysis failed to reveal any gross differences in liver pathology (Fig 5B), we measured AST and ALT in the serum of infected animals as a more sensitive indicator of hepatic injury (Fig 7A). CD1d^-/-^ mice had serum ALT levels that were slightly, yet significantly, lower than those measured in B6 mice, which implied slightly more severe liver damage in the latter strain. However, when taken together with the absence of widespread necrosis, these mild-to-moderate elevations in circulating hepatic enzymes are not consistent with death from liver failure, particularly considering the similar liver burden in the two groups of mice (Fig 5). Such slightly elevated ALT levels need not be a result of the death of infected hepatocytes, but might rather be indicative of increased systemic inflammation [56].

To monitor the numbers of circulating neutrophils we performed complete blood counts (CBC) at various time points post inoculation. B6 mice did indeed show more pronounced neutrophilia d7 p.i. when compared to CD1d^-/-^ mice (Fig 7B). This difference was not observed in naïve mice and did not appear until d7 p.i. (Fig 7C). We found lower G-CSF levels in the serum but not the lungs of CD1d^-/-^ mice by d7 (Fig 7D), which was consistent with the lower frequency of neutrophils in the blood (Fig 7B). Thus, decreased neutrophilia incited by LVS infection in CD1d^-/-^ mice could be one cause for their increased resistance to tularemia.

### Tempered inflammatory response in CD1d^-/-^ mice after LVS infection

**Fig 8:**
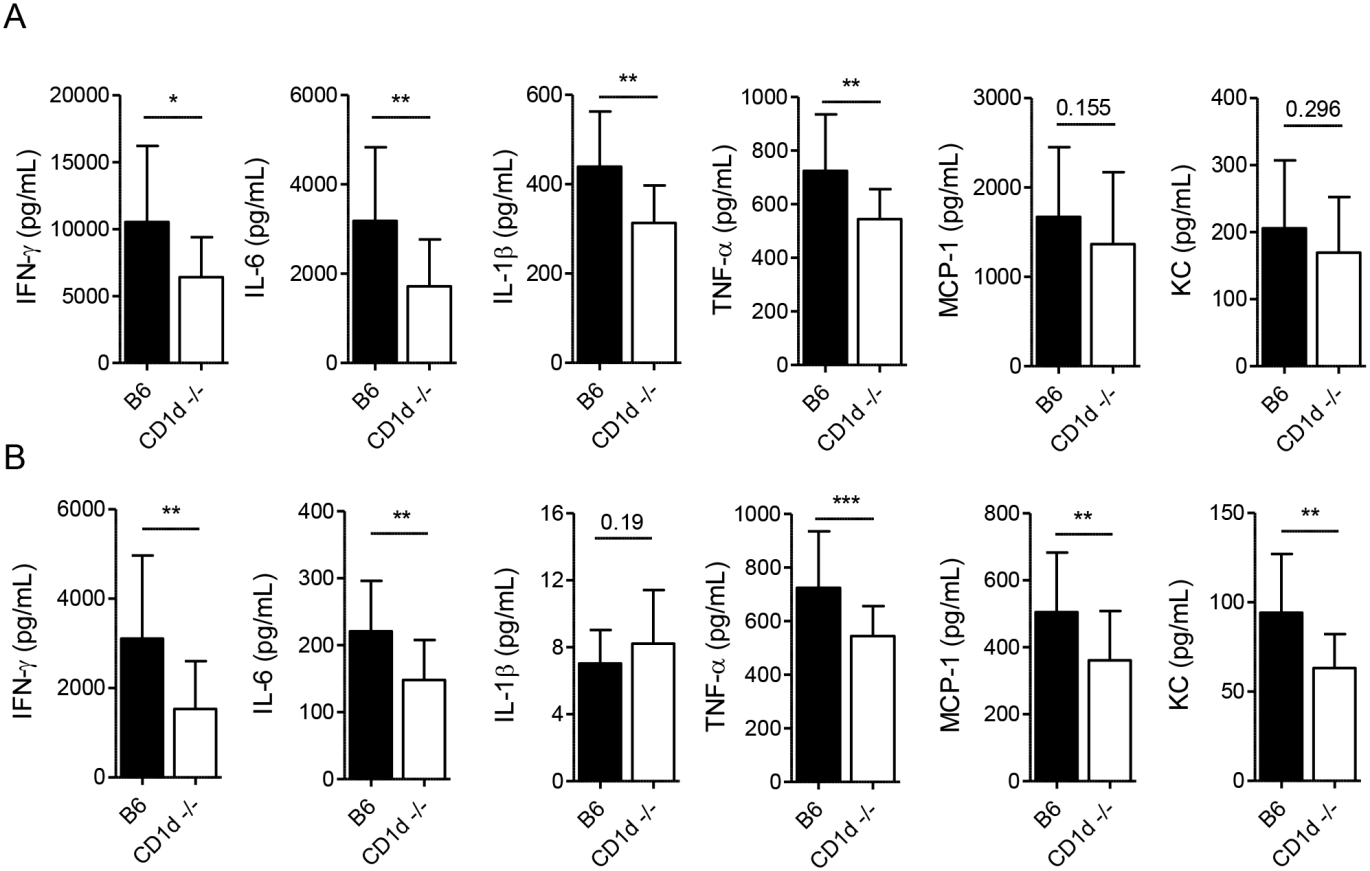
CD1d^-/-^ mice exhibit a tempered inflammatory response to LVS. Cytokine levels were measured at 7d p.i. in lung homogenates **(A)** or serum **(B)** by CBA. Data are combined from at least 3 experiments with 10–20 mice/group. Bars are mean+SD. Comparisons in these experiments were made as indicated in Materials and Methods. *p<0.05, **p<0.01, ***p<0.001.

Although IFN-γ is necessary for resistance to LVS, excessive production of proinflammatory cytokines can be detrimental, particularly after intranasal infection [24,41]. Because NKT cells are known to induce IFN-γ production by NK cells [57,58], we therefore asked whether CD1d^-/-^ mice—which have normal numbers of NK cells—might produce less IFN-γ in response to LVS. We found that although the early IFN-γ response was comparable in both groups of mice (S7 Fig), by d7 p.i. CD1d^-/-^ mice had significantly lower levels in both the serum and lung (Fig 8A and 8B).

Many cytokines produced by NKT cells were suggested to promote sepsis-like inflammatory disease [11,59] and, hence, we measured the levels of those cytokines previously shown to be induced by LVS infection that have also been associated with severe sepsis [49,60]. We found that CD1d^-/-^ mice had consistently lower levels of IFN-γ, IL-6, and TNF-α in both the lungs and serum, with lower levels of MCP-1 and KC in the serum at d7 p.i. (Fig 8A and 8B) which coincided with modestly reduced burden, reduced weight loss, and less severe outward signs of disease (Fig 1). Hence, NKT deficiency results in a less severe “cytokine storm” in response to i.n. LVS infection.

**Fig 9:**
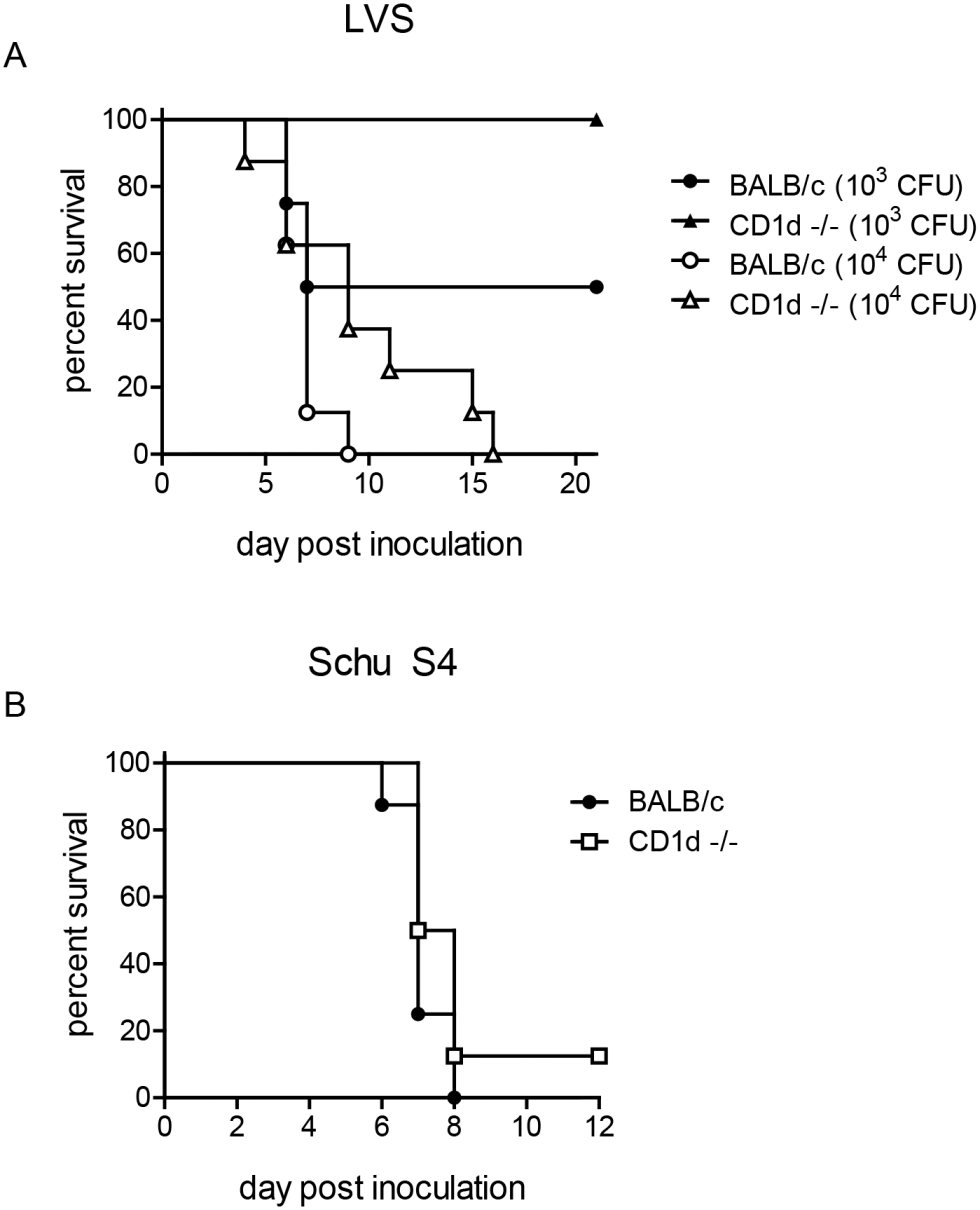
Reduced susceptibility of CD1d^-/-^ mice is not dependent on strain background. **(A)** Groups of 6–8 week-old BALB/c or CD1d^-/-^ mice were infected intranasally with different doses of LVS as indicated and monitored for survival (*n* = 8 mice/group) **(B)** BALB/c or CD1d^-/-^ mice were infected intranasally with 6 cfu Schu S4 and monitored for survival (*n* = 8 mice/group).

Given the observed differences in susceptibility among various mouse strains [61–64], we next ascertained whether CD1d^-/-^ mice in the BALB/c background would exhibit the same phenotype. Consistent with the results in C57BL6 background, we found that CD1d^-/-^ mice were less susceptible to LVS infection in the BALB/c background as well (Fig 9A). As observed with C57BL6 mice, higher doses caused severe disease in both groups, although death was slightly delayed in CD1d-deficient mice.

Mice are highly susceptible to several subspecies of *Francisella* and the extreme virulence of type A (subspecies *tularensis*) or B (subspecies *holarctica*) strains complicates the study of effective immune responses, particularly when administered intranasally (LD_50_<10) [26]. Although LVS has long been used in murine models of experimental tularemia [31,65], it is an attenuated type B strain that fails to cause disease in humans. We therefore sought to determine whether CD1d^-/-^ would have a similar resistance to the more virulent type A strain Schu S4. Not surprisingly however, both wild-type and CD1d^-/-^ mice were extremely susceptible to a low-dose (<10 CFU) pulmonary infection with Schu S4 (Fig 9B), similar to the results obtained with higher doses of LVS in both C57BL6 (S1 Fig) and BALB/c (Fig 9A) mice. These data are consistent with previous reports showing reduced efficacy of therapies or mutations that confer resistance to LVS when challenged with Schu S4 [35,66–68].

## Discussion

Once infected, the host has two strategies to deal with invasive pathogens: resistance and tolerance. Resistance implies the ability of the host to clear a pathogen or limit its spread whilst tolerance indicates the ability of the host to bear the ensuing pathology [69]. Surviving infection from a pathogen such as *F*. *tularensis* likely requires a balance of both. Previous studies of infection in immunodeficient mice revealed that immunity to *F*. *tularensis* is mediated by the concerted effort of the innate and adaptive humoral and cellular immune responses. Because the host deploys a wide array of effector mediators in its inflammatory arsenal against the infection a sepsis-like disease ensues [22,26]. Hence, fatalities in this infectious disease appear to be caused by an inability to control bacterial growth and dissemination, which precipitates inflammation and causes irreparable collateral tissue damage. Accordingly, if diagnosed early, antibiotic treatment limits bacterial burden and tempers inflammation thereby controlling morbidity and mortality underlying tularemia. Herein we report that NKT cells respond to intranasal infection by migrating to the infected lung to produce IFN-γ, which promotes clearance and killing of the pathogen. As a trade-off however, the early activation of NKT cells causes excessive systemic inflammation, increases neutrophil mobilization, and delays infiltration of lymphocytes into the lungs and formation of protective tertiary lymphoid structures. Hence, CD1d-deficient animals have a tempered inflammatory response and perform clinically better in response to intranasal LVS infection. This advantage, however, can be overcome with higher doses of bacteria or infection with more virulent subspecies. The discordant results obtained using Jα18^-/-^ and CD1d^-/-^ mouse models likely reflect a more global T cell deficiency in Jα18^-/-^ mice and underscore the difficulties in deciphering the role of NKT cells using currently available methods [14].

Akin to CD1d-deficient animals, *F*. *tularensis* infection of metalloproteinase-9 null as well as IL-17 and IL-10 double-deficient mice showed low disease incidence despite similar bacterial burden [35,41]. In conjunction with a tempered inflammatory response, these immunodeficient mice were able to limit the damage caused by the pathogen or the host immune system and were therefore less susceptible to i.n. *F*. *tularensis* infection. These findings and our data reported herein, thus enforce the notion that the host response to *F*. *tularensis* is a balance between resistance to pathogen and tolerance to pathology.

LVS infection of endothelial cells induces the transmigration of neutrophils into infected tissues [70,71]. Rather than contributing to host defense, neutrophils may instead increase disease severity. *F*. *tularensis*-infected neutrophils assume an extended proinflammatory phenotype, which is thought to contribute to increased tissue destruction [72]. Because *F*. *tularensis* is able to infect and replicate within neutrophils, these cells likely serve as a vehicle for dissemination from the primary site of infection, which might contribute to the reduced peripheral burden observed in CD1d^-/-^ mice [15,73]. Direct interaction with NKT cells has been shown to influence the function of neutrophils [74] and to promote hematopoiesis in both humans and mice [75]. NKT cell-neutrophil interactions were also recently shown to exacerbate polymicrobial sepsis [59]. Although we found no differences in differential cell counts from peripheral blood of naive wild-type and CD1d^-/-^ mice, CD1d^-/-^ mice displayed less severe neutrophilia upon LVS infection. We also found that slightly decreased hepatotoxicity, reduced peripheral burden, and less severe inflammation in NKT cell-deficient CD1d^-/-^ mice corresponded with less severe neutrophilia.

Our studies revealed that acute LVS infection of CD1d^-/-^ mice resulted in increased lymphocytic infiltration in the lung interstitium when compared to B6 mice. The cellular infiltration organized into iBALT at the peak of infection. Although typically associated with chronic inflammatory conditions, iBALT has been shown to form in the lung in response to viral infection [51]. However, the formation of such structures in response to acute bacterial infection is less well-studied. In the context of pulmonary *F*. *tularensis* infection, lymphoid aggregates have been described in wild-type mice that survived *F*. *tularensis* infection after >50d p.i. whilst bone fide iBALTs were described only in LPS/rPorB vaccinated mice challenged with a high dose of LVS [49,76]. The post-vaccination development of these structures in the lungs was associated with better survival upon i.n. challenge with LVS or the more virulent *F*. *tularensis* subspp. *tularensis* [76,77]. Data from these studies suggest that the T and B cells found in iBALT limit the spread of the bacteria from the lung thereby reducing immune-mediated damage to peripheral organs. Contrary to the results of these studies with wild-type animals, our data demonstrate the development of iBALT in the lungs of B6 mice is hampered perhaps by the presence and activation of NKT cells as more robust iBALT formation is observed in CD1d^-/-^ mice during the peak of a primary LVS infection. Such early iBALT formation is suggestive of a more vigorous and effective adaptive immune response resulting in reduced systemic inflammation and therefore less severe disease. These findings suggest a suppressive/regulatory role for pulmonary NKT cells. The regulatory function of NKT cells is just beginning to be elucidated and much of what we know derives from studies utilizing model antigens [7]. Thus, our findings provide a model in which to study the potentially detrimental functions of iNKT cells in a natural infection.

In the context of intranasal LVS infection, the inability to form iBALT proves to be detrimental, but there may be cases where this outcome is desirable. For example, iBALT formation has been associated with chronic inflammatory conditions such as asthma [78]. Therefore, the discovery of an NKT cell-activating ligand(s) derived from *F*. *tularensis* that can prevent iBALT formation might be of therapeutic benefit in such instances. Similarly, a better understanding of the regulatory function of NKT cells may lead to advances toward the goals of rational vaccine design.

## Materials and Methods

### Ethics statement

All procedures on mice were performed by approval from the IACUC at Vanderbilt University School of Medicine and at Albany Medical College. Anesthesia was performed using 1–5% isoflurane or 100 mg/kg and 10 mg/kg ketamine and xylazine, respectively. Euthanasia was performed by CO_2_ overdose followed by cervical dislocation.

### Mice

C57BL/6J mice were purchased from the Jackson Laboratory (Bar Harbor). CD1d^-/-^ mice were a gift from L Van Kaer (Vanderbilt University School of Medicine). Jα18^-/-^ mice were generously provided by M Taniguchi (RIKEN Institute, Yokohama, Japan). Mice were bred and maintained in the School of Medicine vivarium and provided with food and water ad libitum. LVS infection was performed in an ABSL-2 facility. Vanderbilt’s IACUC approved the experiments described here.

### Bacterial infection

*F*. *tularensis* LVS was provided by S. Khader (Washington University, St. Louis, MO). Preparation of working stocks and CFU determination from infected tissue were performed as described [31]. LD_50_ was determined by the method of Reed and Muench [32]. Male, 8–12-week old mice were anesthetized by i.p. administration of a ketamine/xylazine mixture and ~8–10x10^3^ CFU LVS were administered i.n. in 50μL sterile PBS. Mice were monitored daily for weight loss and signs of morbidity. Criteria for clinical score were developed based upon observation of mice from at least three separate experiments: 1) no outward signs of illness; 2) consistently ruffled fur; 3) hunched back and altered gait; 4) reduced mobility/reaction to stimulus, labored breathing, lethargy. Mice were humanely euthanized when weight loss exceeded 30 percent.

Experiments with Schu S4 were conducted with approval of the Albany Medical College IACUC (protocols 15–04001 and 15–04002). Six-to-eight week old BALB/c and C.129-CD1d^-/-^ mice were inoculated i.n. with the indicated dose of Schu S4 as described above for LVS.

### Cytokine measurements

Cytokines were measured in serum or lung homogenates using Cytokine Bead Array assay (BD Biosciences). Right lobes of lung were homogenized using a Tissue Tearor (Biospec Products, Inc.) in 1 mL sterile PBS containing a cocktail of protease inhibitors (Roche). Homogenates were centrifuged and supernatants frozen at -80°C until analyzed.

### Pulse Oximetry

SpO_2_ was measured using the PhysioSuite with MouseSTAT Pulse Oximeter (Kent Scientific Corp.). Mice were anesthetized using 1.5% isoflurane for induction and maintenance. Sensor was attached to the right hind foot according to the manufacturer’s instructions. Measurements were recorded at 5-second intervals for at least 4 minutes and averaged.

### Tissue processing

Spleen, liver, and lungs were processed as previously described [1,79]. Left lung lobes were used for flow cytometry and microscopy and right lobes for CFU determination.

### Flow cytometry

NKT cells were analyzed as described [79]. All data were acquired on an LSR II (BD Biosciences) and analyzed with FlowJo software (FlowJo, LLC). Cell populations were identified as follows: B cells (CD19^+^); T cells (CD3ε^+^); NK cells (CD3ε^-^NK1.1^+^); NKT cells (CD3ε^+^CD1d/αGC tetramer^+^); DCs (CD45^+^CD64^-^CD24^+^MHCII^+^CD11c^+^). DC subsets were identified as described [80]. CD1d monomers were provided by the NIH Tetramer Core Facility. Cell counts were determined using AccuCheck counting beads (Invitrogen).

### Immunofluorescence and confocal microscopy

To analyze the formation of tertiary lymphoid follicles in the lungs following LVS infection, tissues were fixed in PLP buffer (2% paraformaldehyde, 0.05 M phosphate buffer containing 0.1 M l-lysine, and 2 mg/ml NaIO_4_) overnight. Tissues were then dehydrated in 30% sucrose and subsequently embedded in OCT media. Twenty micron frozen sections were preincubated with Fc-block (anti-mouse CD16/32 Ab; Biolegend) diluted in PBS containing 2% goat serum and fetal bovine serum (FBS). After incubation for 1 hour at room temperature, sections were washed with PBS and stained with the following antibodies diluted in PBS containing 2% goat serum and FBS for 1 hour at room temperature: anti-B220-Alexa-488 (Biolegend), anti-CD3-allophycocyanin, anti-CD11c-phycoerythrin (Biolegend) and Fc-block. Sections were washed with PBS and mounted using Immu-Mount (Thermo Fisher Scientific). Images were acquired using a Zeiss 780 confocal microscope (Carl Zeiss). The imaging data were processed and analyzed using the Imaris software (Bitplane; Oxford Instruments).

### Statistical analysis

Statistical analyses of representative data were performed using GraphPad Prism version 5.02 for Windows, GraphPad Software, San Diego California USA (www.graphpad.com). Where indicated in the figure legends, data were aggregated across several (from two to four) replicate experiments. To address the clustered nature of the final datasets, we used a linear regression analysis with cluster robust standard error estimation (an extension of Huber-White Sandwich Estimator [81–83]), a method that accounts for intraclass correlation when determining statistical significance of regression coefficients. In all reported regression analyses individual data points were entered as the dependent variable, and replicate experiments were identified as the clustering variable. Experimental group type (KO and WT) was entered as the independent variable and was dummy coded for the analyses with “KO” group as the reference category. In such case, the intercept reflects the mean of the reference group (= KO), and the slope reflects the difference between the KO and the WT groups. We have reported herein the significance of coefficients for slopes (differences between group means). Clinical score data was analyzed using Generalized Estimating Equation (GEE) model based on Poisson regression. In the model, clinical score was predicted by group type and day of assessment (dummy coded), and their interaction. We report comparisons between groups at each time of assessment, which were obtained by changing the reference category between days of assessment. All analyses were carried out using R Project for Statistical Computing (http://www.r-project.org/).

## Supporting Information

S1 Fig **(A) Groups of B6 mice were inoculated intranasally with increasing doses of LVS as indicated and monitored daily for weight loss and signs of morbidity.** Data are representative of two similar experiments with 5 mice/group. Plotted are mean ± SD. (B-D) Groups of B6 or CD1d^-/-^ mice were inoculated intranasally with 5 x 10^2^ (B), 2 x 10^3^ (C), or 3 x 10^4^ (D) cfu LVS and monitored daily for signs of morbidity. Mice were humanely euthanized when weight loss exceeded 30% or when showing obvious signs of distress. Data are representative of two or three experiments with 8–10 mice/group.
(TIF)

S2 Fig Increased NKT cell numbers exacerbates disease. Groups of WT or Vα14^tg^ mice were infected intranasally with 8000 cfu LVS and monitored daily for weight loss and signs of morbidity (n = 6–9 mice/group). Results are representative from one of three similar experiments.
(TIF)

S3 Fig NKT cells are pre-positioned in the lung and recruited into the interstitium after i.n. LVS inoculation. **(A)** Representative plots of lung lymphocyte localization in naïve B6 mice. Cells were identified as described in Materials and Methods. Intravascular αCD45 staining was used to discriminate intravascular (αCD45^POS^) and interstitial (αCD45^NEG^) cells. Numbers are percent of each cell type within the respective gate. **(B)** Representative intravascular staining of NKT cell localization d3 after intranasal administration of 2 μg αGalCer (top) or ~8,000 cfu LVS (bottom). Numbers are percent of CD3ε^+^CD1d/αGalCer tetramer^+^ cells. **(C)** Representative NKT staining of blood from mock- or LVS-infected mice at various time points p.i. Numbers in plots are percent of B220^-^ lymphocytes.
(TIF)

S4 Fig Lung burden, but not liver or spleen, are correlated with weight loss after i.n. LVS infection. LVS burden was determined from homogenized lung, liver, and spleen d7 p.i. Data are cumulative from more than three experiments with *n* values as indicated. Spearman correlation analysis showed that only lung burden was correlated with weight loss at the peak of infection.
(TIF)

S5 Fig Tertiary lymphoid structures are more prominent in lungs of NKT-deficient mice. Representative sections from B6 (left) and CD1d^-/-^ (right) mice d7 post i.n. inoculation (8,000 cfu LVS).
(TIF)

S6 Fig iBALT is not observed in the lungs of naïve, uninfected mice. Representative images of naïve lung sections from B6 (left) or CD1d^-/-^ (right) mice.
(TIF)

S7 Fig CD1d^-/-^mice have an early IFN-γ response that is comparable to B6 mice. Lung and serum IFN-γ levels were determined in naïve mice or at various time points p.i. as in Fig 8. Data are combined from 3 independent experiments (*n* = 15 mice/group). Plotted are mean±SD.
(TIF)
