# Supplementary figures and images for: Border Patrol Gone Awry: Lung NKT Cell Activation by Francisella tularensis Exacerbates Tularemia-Like Disease
Source: PLoS Pathog. 2015 Jun 11;11(6):e1004975. doi: 10.1371/journal.ppat.1004975 (PMC4465904; doi:10.1371/journal.ppat.1004975)

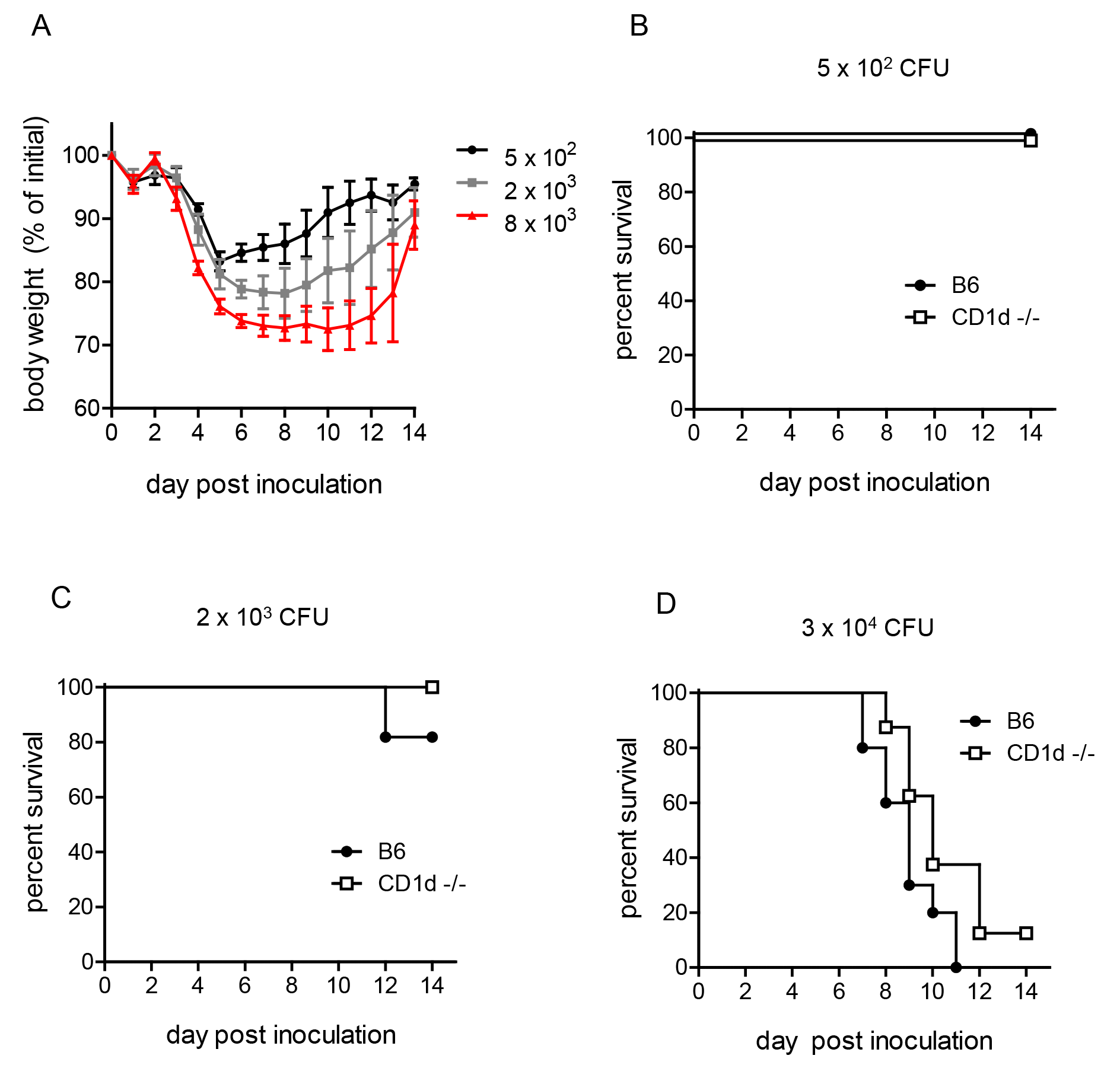

Supplement: S1 Fig — (A) Groups of B6 mice were inoculated intranasally with increasing doses of LVS as indicated and monitored daily for weight loss and signs of morbidity. Data are representative of two similar experiments with 5 mice/group. Plotted are mean ± SD. (B-D) Groups of B6 or CD1d-/- mice were inoculated intranasally with 5 x 10^2^ (B), 2 x 10^3^ (C), or 3 x 10^4^ (D) cfu LVS and monitored daily for signs of morbidity. Mice were humanely euthanized when weight loss exceeded 30% or when showing obvious signs of distress. Data are representative of two or three experiments with 8–10 mice/group. (TIF) [file ppat.1004975.s001.tif]

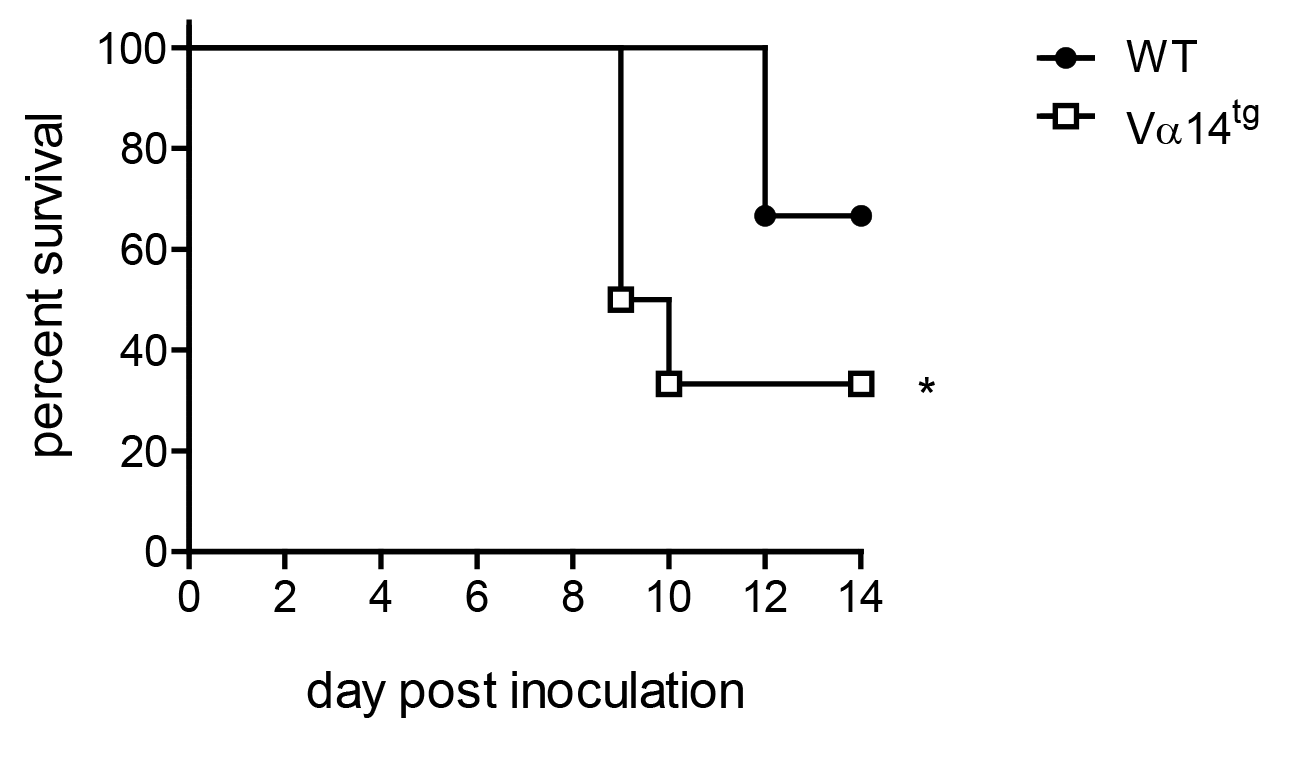

Supplement: S2 Fig — Groups of WT or Vα14tg mice were infected intranasally with 8000 cfu LVS and monitored daily for weight loss and signs of morbidity (n = 6–9 mice/group). Results are representative from one of three similar experiments. (TIF) [file ppat.1004975.s002.tif]

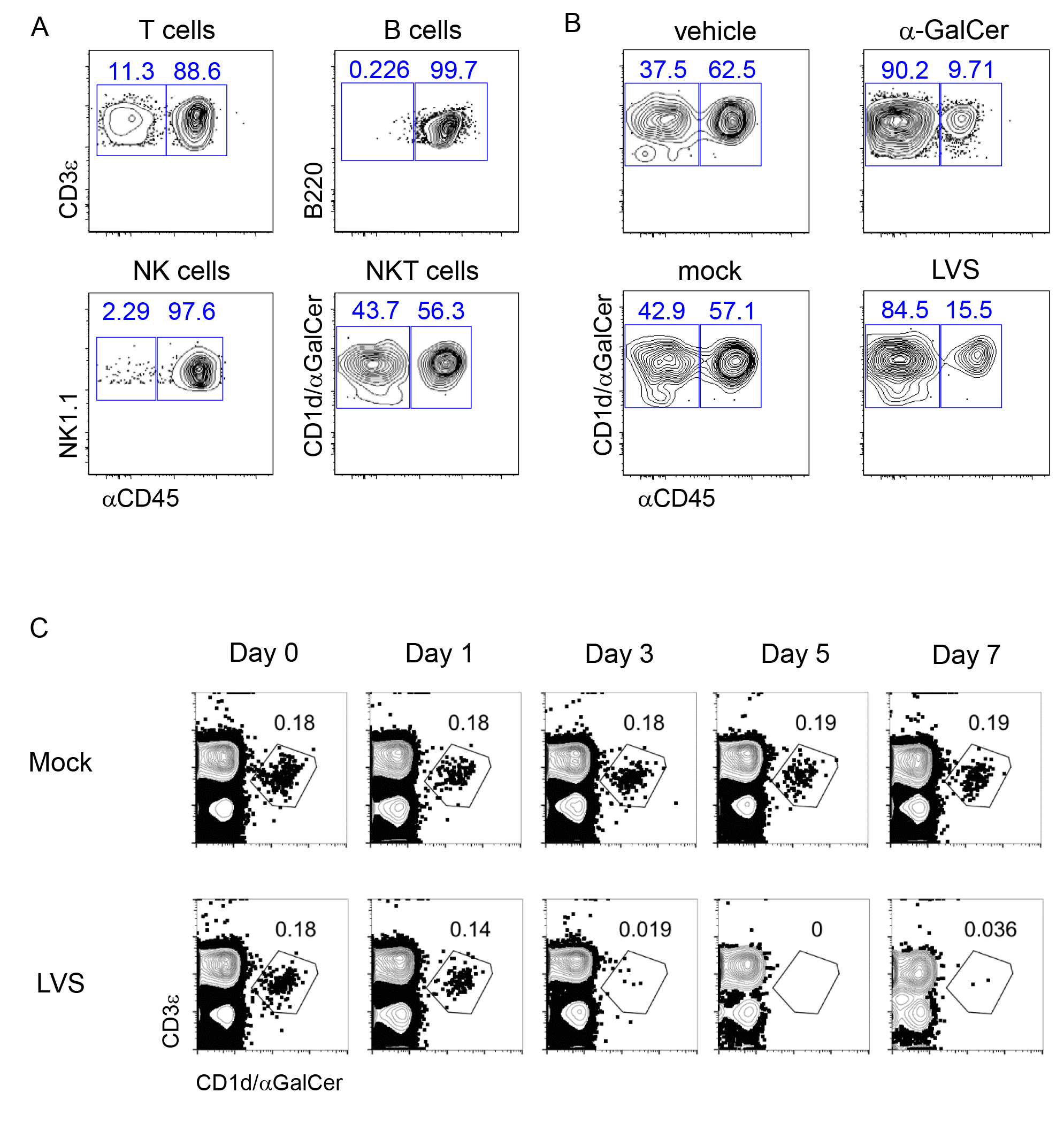

Supplement: S3 Fig — (A) Representative plots of lung lymphocyte localization in naïve B6 mice. Cells were identified as described in Materials and Methods. Intravascular αCD45 staining was used to discriminate intravascular (αCD45POS) and interstitial (αCD45NEG) cells. Numbers are percent of each cell type within the respective gate. (B) Representative intravascular staining of NKT cell localization d3 after intranasal administration of 2 μg αGalCer (top) or ~8,000 cfu LVS (bottom). Numbers are percent of CD3ε+CD1d/αGalCer tetramer+ cells. (C) Representative NKT staining of blood from mock- or LVS-infected mice at various time points p.i. Numbers in plots are percent of B220- lymphocytes. (TIF) [file ppat.1004975.s003.tif]

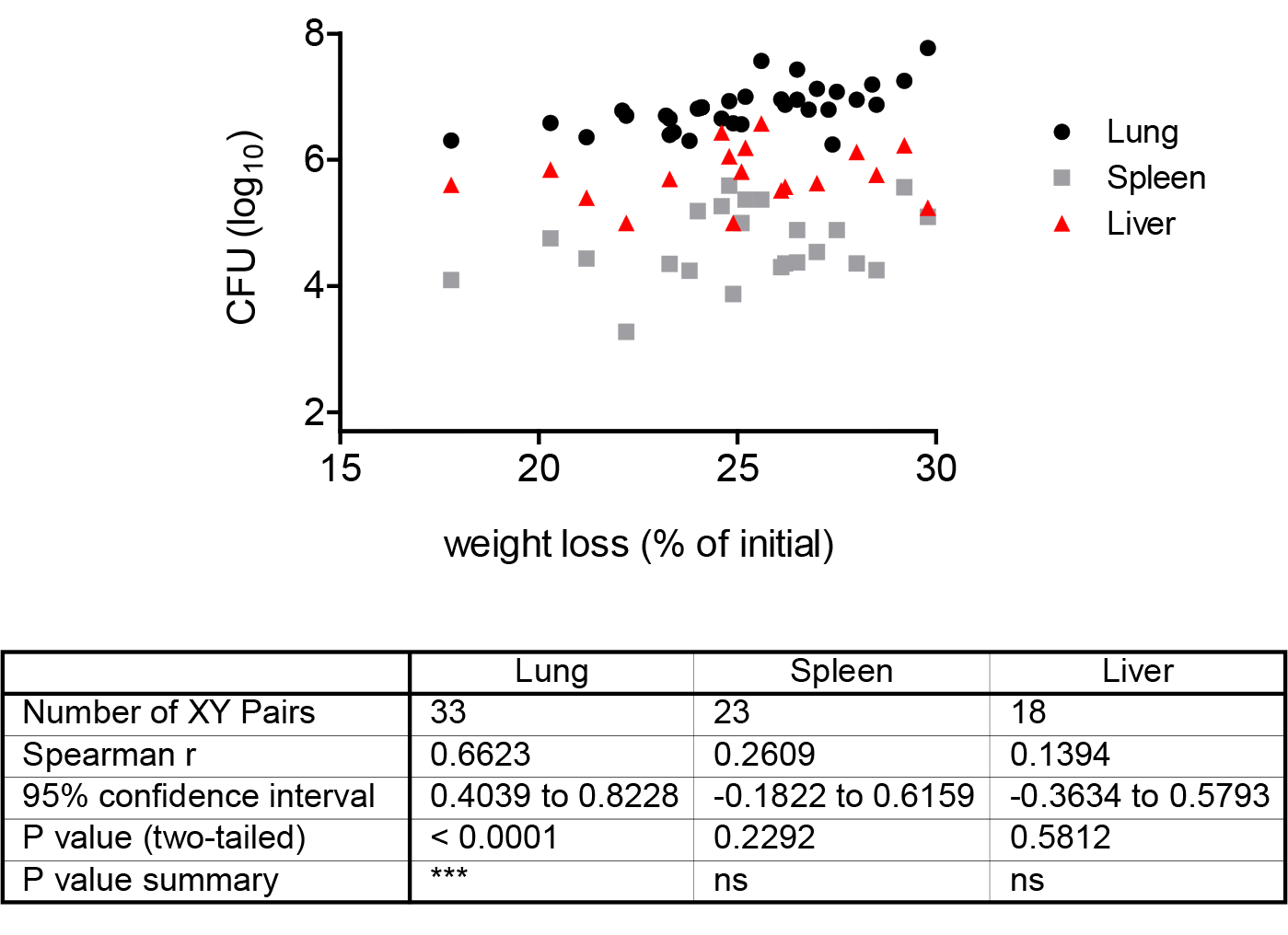

Supplement: S4 Fig — LVS burden was determined from homogenized lung, liver, and spleen d7 p.i. Data are cumulative from more than three experiments with n values as indicated. Spearman correlation analysis showed that only lung burden was correlated with weight loss at the peak of infection. (TIF) [file ppat.1004975.s004.tif]

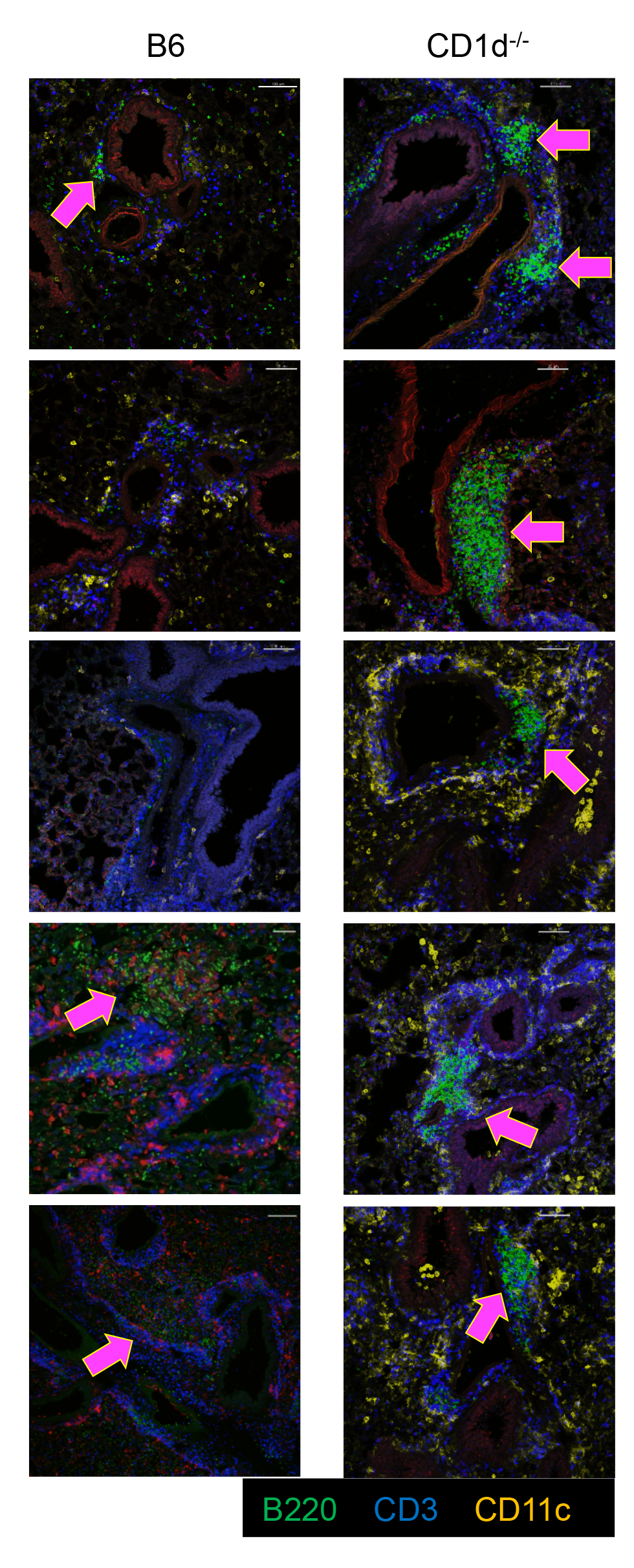

Supplement: S5 Fig — Representative sections from B6 (left) and CD1d-/- (right) mice d7 post i.n. inoculation (8,000 cfu LVS). (TIF) [file ppat.1004975.s005.tif]

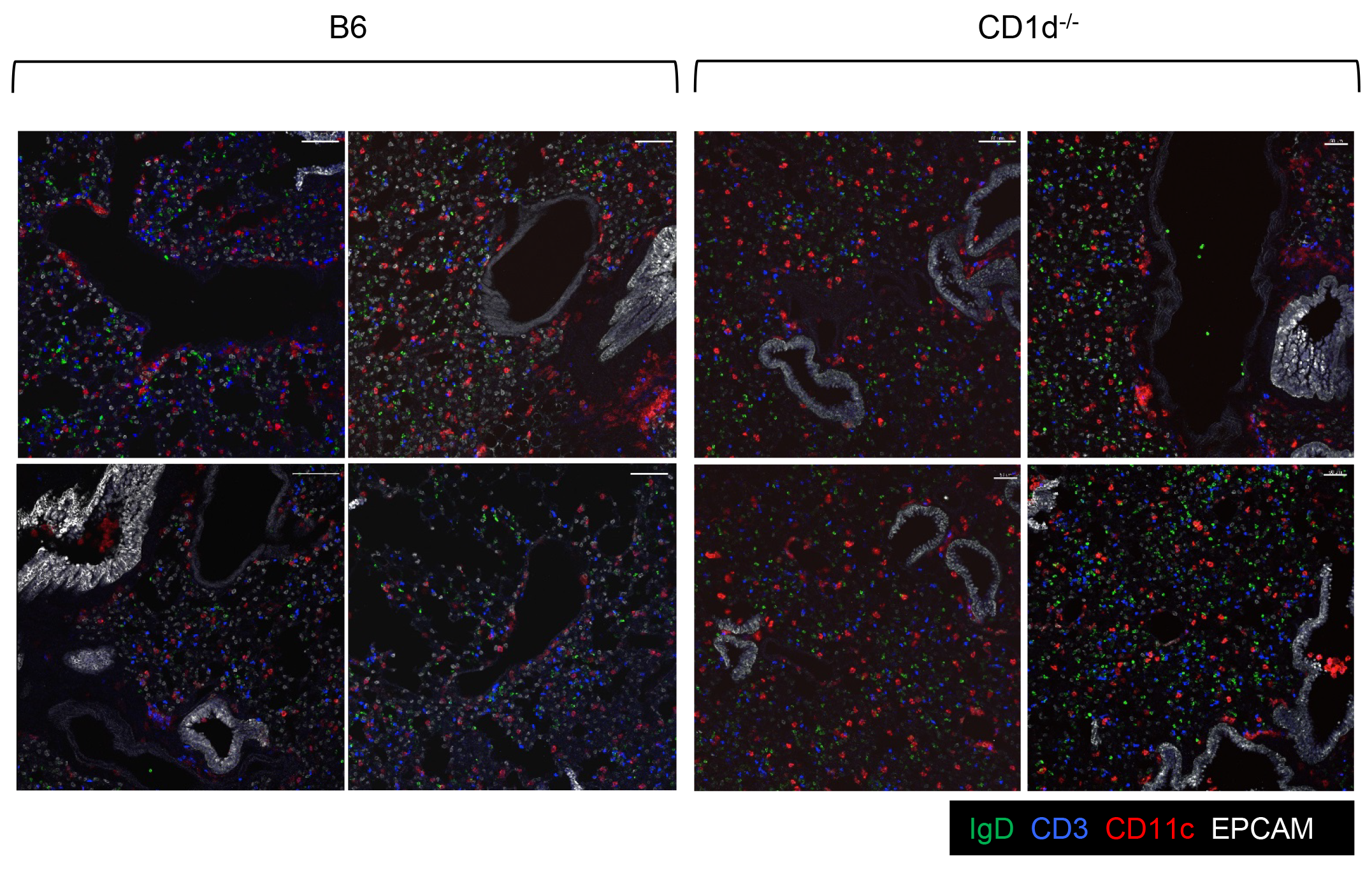

Supplement: S6 Fig — Representative images of naïve lung sections from B6 (left) or CD1d-/- (right) mice. (TIF) [file ppat.1004975.s006.tif]

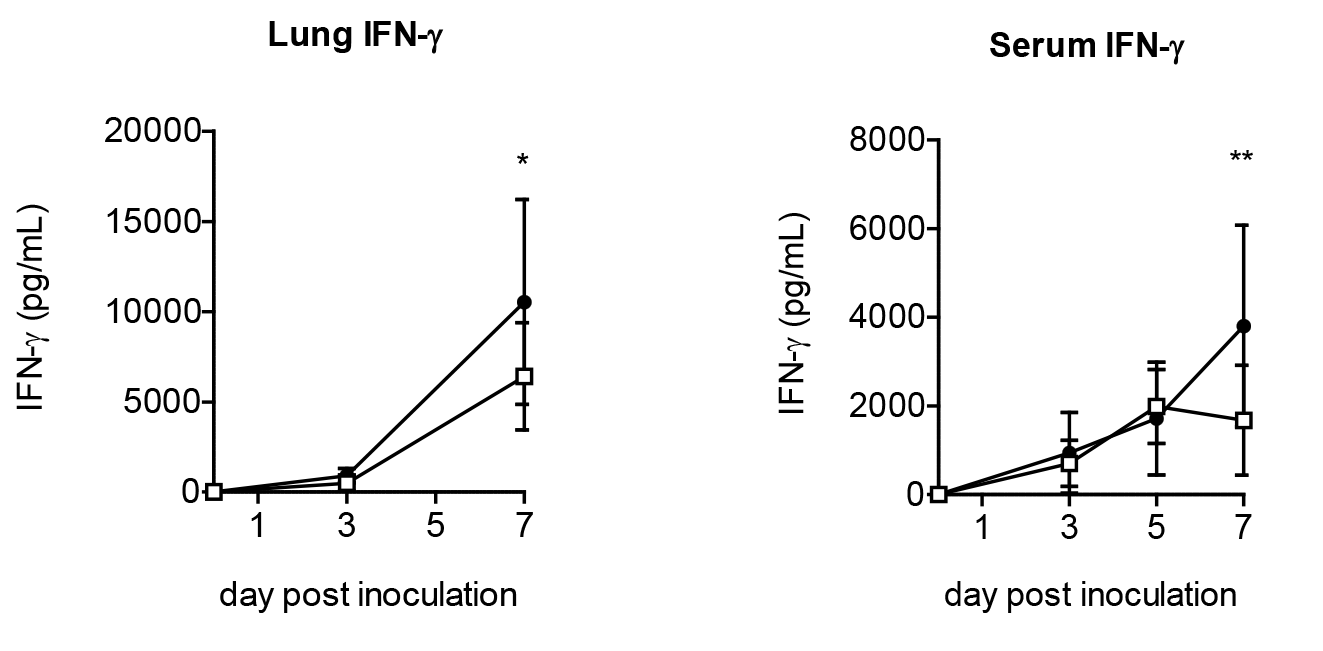

Supplement: S7 Fig — Lung and serum IFN-γ levels were determined in naïve mice or at various time points p.i. as in Fig 8. Data are combined from 3 independent experiments (n = 15 mice/group). Plotted are mean±SD. (TIF) [file ppat.1004975.s007.tif]
